# Stress and Alterations in the Pain Matrix: A Biopsychosocial Perspective on Back Pain and Its Prevention and Treatment

**DOI:** 10.3390/ijerph15040785

**Published:** 2018-04-18

**Authors:** Pia-Maria Wippert, Christine Wiebking

**Affiliations:** 1 Sociology of Health and Physical Activity, Department of Health Science, University of Potsdam, Am Neuen Palais 10, House 12, 14469 Potsdam, Germany; christine.wiebking@uni-ulm.de; 2 Department of Health Sciences and Technology, ETH Zürich, HCP, Leopold-Ruzicka-Weg 4, CH-8093 Zürich, Switzerland; 3 Institute of Psychology and Education, Applied Emotion and Motivation Research, Ulm University, 89081 Ulm, Germany

**Keywords:** stress, allostatic load, relaxation, back pain, chronic pain, physical activity, exercise, neuroplasticity, pain matrix

## Abstract

The genesis of chronic pain is explained by a biopsychosocial model. It hypothesizes an interdependency between environmental and genetic factors provoking aberrant long-term changes in biological and psychological regulatory systems. Physiological effects of psychological and physical stressors may play a crucial role in these maladaptive processes. Specifically, long-term demands on the stress response system may moderate central pain processing and influence descending serotonergic and noradrenergic signals from the brainstem, regulating nociceptive processing at the spinal level. However, the underlying mechanisms of this pathophysiological interplay still remain unclear. This paper aims to shed light on possible pathways between physical (exercise) and psychological stress and the potential neurobiological consequences in the genesis and treatment of chronic pain, highlighting evolving concepts and promising research directions in the treatment of chronic pain. Two treatment forms (exercise and mindfulness-based stress reduction as exemplary therapies), their interaction, and the dose-response will be discussed in more detail, which might pave the way to a better understanding of alterations in the pain matrix and help to develop future prevention and therapeutic concepts.

## 1. Introduction

Chronic pain is one of the most disabling health complaints, with the highest disability rate worldwide [1]. The majority of these complaints are caused by back pain and arthritis [2]. Given the fact that low back pain (LBP) has a high lifetime prevalence (84%) and there is a significant risk of subsequent chronification after the first onset of the disease [2,3,4], research into the underlying mechanisms of these types of chronic pain is of significant interest. Similarly, sophisticated risk assessments, effective prevention strategies, and therapeutic treatments need to be developed to minimize the need for healthcare and the consequent societal economic burden. In line with the multifaceted character of chronic pain, these concepts need to consider musculoskeletal, neuronal, psychobiological, and etiological factors. On the one hand, pain symptoms may be associated with pre-existing vulnerabilities and structural pathologies. On the other hand, functional and/or physical difficulties can be triggered by psychosocial and psychophysiological factors such as stress [5].

Therefore, the objective of this paper is to raise a discussion of probable interacting pathways between physical stress (here defined as PS, i.e., exercise), psychological stress (here defined as MS, i.e., mental stress) and their potential neurobiological consequences in the genesis and treatment of chronic pain. In order to focus on treatment interventions that are easy to learn, inexpensive, and can be integrated in everyday life, two therapy forms—namely physical exercise and mindfulness-based stress reduction—and their interaction and dose-response will be considered in more detail.

## 2. Neurobiological Consequences of Pain

**Figure 1:**
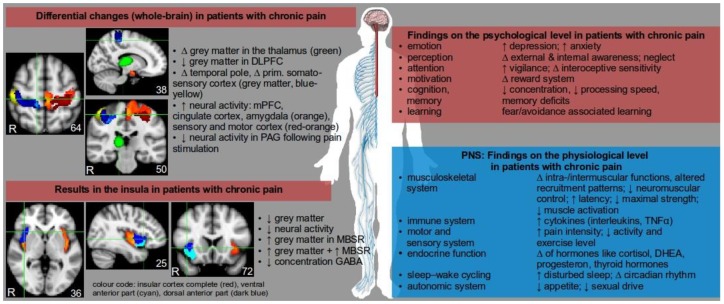
Overview of the complex and multidimensional changes in the central (red area) and peripheral nervous system (PNS, blue area) in patients with chronic pain. ↑ increased, ↓ decreased, ∆ change, + positive relation. Abbreviations: ACTH = adrenocorticotropic hormone, DHEA = dehydroepiandrosterone, GABA = γ-aminobutyric acid, MBSR = mindfulness-based stress reduction, PAG = periaqueductal grey, PFC = prefrontal cortex, TNF = tumor necrosis factor. The content of the picture is not exhaustive, but serves to demonstrate the complexity of chronic pain by listing relevant consequences, cofactors, and epiphenomena. Brain regions displayed on MNI template.

Chronic pain is associated with diverse symptoms and severity, and both functional and structural changes in the central nervous system (CNS) as observed in recent neuroimaging studies (see Figure 1). Pain signals are processed in different brain regions and affect various brain networks, and the term “pain matrix” has been established to describe three major systems commonly affected by pain signals: the lateral and the medial system as the two main afferent pain pathways, and the descending system involved in pain perception [6,7]. Due to the vast efferent and afferent connections of thalamic nuclei it was assumed that chronic back pain patients may show alterations in these subcortical areas.

Using structural magnetic resonance imaging (sMRI) to investigate structural changes in the brains of chronic back pain patients provided inconsistent results. Contradictory findings revealed both an increase [8] and a decrease [9] of grey matter volume in the thalamus of chronic back pain patients. However, two studies reported a reduction of grey matter volume in the dorsolateral prefrontal cortex (DLPFC), though other MRI results indicated opposite findings. A recent meta-analysis [10] confirmed structural changes in chronic back pain patients, particularly in the dorsolateral prefrontal cortex (DLPFC), temporal lobe, the insular cortex, and the primary somatosensory cortex (see also the paper by Baliki et al. [11]). Variations in the white matter between patients with chronic back pain and controls have been investigated less frequently, which makes drawing a conclusion about neurobiological structural changes in these populations difficult at the present time. In the future, methodological imaging problems like the influence of pain medication (such as analgesic drugs and antidepressants) on blood flow and pressure need to be considered, as these factors may have a substantial impact on blood-oxygen level dependent signals [12].

In contrast to the quantification of grey matter changes investigated by sMRI, results of neuroimaging studies using resting-state functional MRI (rsfMRI) in chronic back pain patients show more consistent results. Increased neural activity in chronic pain patients during resting-state fMRI was reported in various cortical brain regions such as the medial prefrontal cortex (MPFC), cingulate cortex, or the insular cortex, as well as in subcortical brain regions like the amygdala [13]. In addition, sensory and motor cortex areas, which play a major role in pain perception and chronification processes, showed increased neural activity and connectivity to other areas of the brain (see Figure 1). It can further be assumed that a causal relationship exists between cortico-striatal connectivity and pain persistence [11]. Compared to studies using resting-state fMRI, there are few studies investigating neural changes in chronic back pain patients using stimulus-induced fMRI; that is, during the application of painful pressure stimuli. Stimulus-induced fMRI studies in chronic pain patients are difficult to conduct due to ethical concerns and movement-related artefacts in fMRI analysis. Moreover, such neuroimaging studies differ in terms of patient population (chronic back pain, fibromyalgia, rheumatism, etc.), applied stimuli (electrical shock, heat stimuli, pressure, etc.), location of applied stimuli (fingernail, forearm, abdomen, etc.), intensity of applied pain (ranging from unexpected versus expected and/or ambiguous light pain to intense pain), imaging methods (fMRI, sMRI, rsfMRI, electroencephalography, etc.) and analysis techniques (voxel-based morphometry, multivariate pattern analysis, functional connectivity, regional homogeneity, etc.). Despite these multiple approaches each characterized by certain advantages and disadvantages, several results indicate a rather consistent pattern of neural activity relating to pain: increased activity in the somatosensory cortex and the insular cortex after painful stimulation. However, patients with chronic back pain showed decreased neural activity after painful stimulation in the periaquaductal grey [14]. Since the insular cortex plays a central role in physiological regulation processes, body-related focused attention, and pain processing, differences in neural activity, connectivity, and structural changes might play a role when considering the potential of therapies like mindfulness-based stress reduction (MBSR, see Figure 1, e.g., Paulus’ research [15]), amongst other psychological and cognitive-behavioral approaches.

The reported neurobiological alterations in the imaging studies are based on prolonged sensitization processes in the peripheral and central pain processing components of the CNS. Whilst acute pain stimuli in the periphery lead to activation of nociceptors on peripheral nerve fibres, processed by mechano-, chemo- or thermo-receptors, different types of peripheral and central neurotransmitters such as serotonin, the substance P, histamine, and prostaglandins are involved in signal transmission. These neurochemical mediators interact with each other and can trigger reorganization processes in nociceptors, such as increased recruitment of receptors and sensitization towards painful stimuli. Finally, modulatory changes constitute increased afferent activity to a given painful stimulus due to inflammation being present, a phenomenon called primary hyperalgesia [16]. Prolonged abnormal afferent information from pain receptors is relayed to the spinal cord (via neurons of the first lamina of the posterior grey horns) and transferred to supraspinal brain areas. Amongst others, ascending pain pathways project to the reticular formation in the brainstem (involved in somatic motor and cardiovascular control and pain modulation), the limbic system (including the anterior cingulate cortex; this system is involved in affective-cognitive aspects of pain including anxiety, emotion, and memory) and the somatosensory cortex (determination of location, intensity, and duration of painful stimuli). Conversely, these brain areas influence descending antinociceptive pathways by modulating descending serotonergic and noradrenergic signals from the brainstem that regulate nociceptive processing at the spinal level. Thus, spinal sensitivity in response to peripheral pain stimuli is modulated by a complex network of inhibitory and excitatory spinal and brain circuits, linking the modulation of the descending system to the level of cord sensitivity in a behavioural and environmental context. For example, the experience of excessive and prolonged physical pain can cause feelings of anxiety and sleep deficits, and—as a result of this reciprocal regulation—the sensation of pain becomes more intense [16].

Taken together, the long-term increase in pain sensitivity is associated with abnormal signal transmissions within the spinal cord and changes in the descending pathway involved in the regulation of pain. The resulting hyperexcitability to stimuli can occur at both the peripheral and central level; for example, minor peripheral signals may evoke a severe experience of pain if the self-reinforcing mechanism (so-called “wind-up”) is established centrally.

Although this reciprocal pain regulation is well-documented [16], our knowledge about the genesis of chronic pain syndromes and effective treatments is still limited. Whereas pharmaceutical approaches promote solutions within the scope of inhibitor identification (e.g., steroids/nonsteroidal anti-inflammatory drugs (NSAIDs), serotonin or noradrenaline reuptake inhibitors (SSRIs, SNRIs), etc.), the behavioural and exercise sciences investigate the effects of conditioning principles and adaptation. From this point of view, the experience and perception of pain, including regulatory pain mechanisms, may go well beyond neural networks, nerve cells, and neurochemical factors. Various other factors need to be considered in the framework of chronic pain as it is moderated by age, gender, psychosocial factors, lifestyle conditions, and genetic *predispositions* [16].

## 3. Neurobiological Consequences of Physical Stress (PS) Such as Exercise

### 3.1. Adaptation to Exercise in the Prevention of Chronic Pain

Neural plasticity of the nervous system is essential in order to accomplish neurobiological adaption to physical activity and exercise, which are important players in the prevention of chronic pain. The central nervous system is responsible for a stable foundation of extremity movements, co-contraction of particular muscles, appropriate muscle recruitment, and the adaptability and timing needed for core stability [17]. Altered muscular interaction schemes or altered afferent combinations, such as inhibition or aberrant innervation of individual muscles, dramatically affect spinal statics, movement execution, and spatial constancy mechanisms. Furthermore, altered phasic sequences of synergistic muscle activations can affect the stability of trunk and limb movements. This can be compensated for by motor control exercises for trunk muscles aimed at improving stability, mobility, intra and intermuscular function, and innervation patterns. However, the general advantage of exercise may additionally be based on neuroplastic central effects. This applies to central pain-processing areas and induces changes in cell growth, vascularization, and morphologic alterations of grey matter [18,19,20]. These intended positive exercise effects may prohibit the described pain-induced maladaptive changes in brain structures (see Section 2). Another interesting effect of most types of exercise is a reduction of pain perception following acute exercise, a phenomenon known as exercise-induced hypoalgesia (EIH) [21,22]. Although there is evidence that exercise induces a higher pain tolerance [23], the appropriate exercise dose for an optimal EIH response is still unknown [24,25]. This lack of knowledge limits the systematic application of the EIH effect to therapy programs [22] despite it being proven as effective in some studies [26]. Further, the mechanisms of EIH are not fully understood; on the one hand, study results imply that exercise may be related to a reduced endogenic pain inhibition system caused by altered processing of somatosensory stimuli [27]. On the other hand, some studies suggest an involvement of opioid and non-opioid mechanisms [21,28]. However, both of the main effects of exercise (central and peripheral) affect the reciprocal regulation sides in the genesis of chronic pain and may be useful in preventive strategies (see Section 2).

It is particularly important to emphasize that low back pain patients show a variety of central and peripheral maladaptations: studies report inadequate constancy, altered neuromuscular activity, and reduced neuromuscular control function as well as increased latency and reduced activation under physical stress and perturbation [17,29]. Further, the ability to engage maximum strength and endurance, essential for the compensation of high loads, is also impaired [30].

### 3.2. Adaptation to Exercise in Therapy

Exercises of the trunk muscles are involved in state-of-the-art treatments in the prevention and therapy of low back pain [31,32]. Motor control exercises (sensory-motor training, or SMT), including balancing and strengthening elements, are especially appropriate for improvement in neuromuscular control, strength, and kinematics of the trunk muscles and spine balance during changing mechanical disruptions. However, reviews show that sensory-motor training [33] or other exercises types [34] are not inevitably related to a prolonged pain or disability reduction. An adequate explanation for this has not yet been found.

The search for an exercise treatment that effectively reduces pain symptoms leads to a discussion about the specificity [33], intensity, and dose of an offered exercise [35] that may regulate the dose–response relationship of the pain experience in the individual [22]. Considering the “wind-up” (mechanism of sensitization and reciprocal regulation of pain stimuli, see Section 2), two scenarios can be considered: Firstly, exercises executed at high intensity may induce the activation of afferent pain receptors (located in skin, muscles, and bones). Thus, peripheral sensitization might be further provoked by excessive mechanical load or pre-existing functional deficits. Imaging studies investigating stimulus-induced pain show that peripheral sensitization can be associated with central hypersensitivity, a reduced endogenous pain inhibition [36], and a higher intrinsic activity in the somatosensory cortices with a synchronization of the sensory-motor neural network (SMN) [37]. Secondly, central hypersensitivity in the limbic system, relating to affective-cognitive aspects of pain, influences descending antinociceptive pathways [16]. Depending on the type of exercise (e.g., lacking postural control), anxious feelings, fear of pain, and negative expectations of the exercise treatment may arise, thus resulting in increased pain perception. Both exercise conditions (too intensive/without postural control) would prohibit the intended EIH. To date, the interactions between EIH, fear conditioning, and the dose–response of exercise are still unknown, although they have been raised in proof of concept studies [22,26]. Besides the choice of the right exercise dose, these findings raise a question regarding whether conditioning tasks could affect certain brain areas (such as the limbic system), subsequently interrupting the dysfunctional reciprocal circle, and whether they could therefore be combined with exercise tasks in therapeutic programs.

Evidence from different scientific fields suggests that distraction tasks can be utilized as a deconditioning tool for pain and for disruption of the affective-cognitive mechanisms of fear avoidance to exercise. For example, cognitive distraction is processed in similar cortical networks as the sensory aspects and inhibition of pain (e.g., in the hippocampus, prefrontal cortex, and amygdala [30]) and has been associated with a reduced subjective pain perception in various studies [38]. This is based on the assumption that simultaneous processing of pain signals and cognitive stimuli is limited due to an overlapping and restricted set of mental resources [39]. The desired outcome—reduced subjective pain intensity and increased pleasantness—was observed in several studies, especially for executive memory working tasks (e.g., n-back task [40], Stroop task [41]) or for arithmetic tasks such as the paced auditory serial addition test [40,42]. Currently, a combination of SMT exercise and cognitive tasks has been evaluated in proof of concept studies on low back pain [43,44]. These studies aim for a reduction in the subjective pain experience and increased motor amplitude, as well as an improvement in muscle function and strength, following the ideas of new effective treatment forms and prevention tasks.

## 4. Neurobiological Consequences of Mental Stress (MS)

### 4.1. Adaptation to Psychosocial Stress

In addition to physical stress, biological stress responses can also be induced by psychosocial stressors (here defined as mental stress). This response is driven by the autonomic nervous system (ANS), the anterior pituitary glands, and the hypothalamus [45], whereby the stress response within the ANS proceeds to one of three peripheral catecholamine systems with a release of neurotransmitters (i.e., norepinephrine, acetylcholine, dopamine, or cytokines). Within the anterior pituitary glands and the hypothalamus, the stress response is driven by synaptic information from different brain regions like the limbic system (including the hippocampus and amygdala) or the brainstem (including the locus coeruleus). The effects of the stress response are reflected across five endocrine axes: the hypothalamic-pituitary-adrenal (HPA), hypothalamic-pituitary-thyroid (HPT), hypothalamic-pituitary-gonadal (HPG), hypothalamic-pituitary-somatotropic (HPS), and hypothalamic-pituitary-prolactin (HPP). The major role of these complex response systems is the maintenance of homeostasis and promoting adaption to external and internal challenges [46]. A repeated (long-term) activation of these regulatory systems can lead to pathological changes and an accumulated biological burden on the body. This so-called allostatic load can affect the function of the hypothalamic-pituitary axes (e.g., HPA hyper or hypofunction), and the immune, cardiovascular, and metabolic systems [47]. During the development of an increased allostatic load, other components from the neuroendocrine and immune system play a role, which in turn are associated with stress-related pain disorders [48].

Considering the sensitization process and reciprocal regulation of pain stimuli (“wind up” mechanism), the following scenarios can be assumed for the association between MS and pain: Firstly, microstructural damage [49], musculoskeletal disorders, and local muscle tension reactions [50] may be stabilized in response to altered neurotransmitter concentrations (inflammatory mediators like cytokines, growth hormones and others, see Figure 1) and their peripheral influence on nociceptor-sensitization, receptor recruitment, and signal transmission to the somatosensory cortex. Thus, maladaptive muscle tension can activate afferent pain fibres in the muscles and bones of neighbouring regions and promote peripheral sensitization processes [51]. Secondly, altered neurotransmitter concentrations in supraspinal brain areas caused by HPA-hypofunction constrain descending antinociceptive pathways by modulation of descending serotonergic and noradrenergic signals from the brainstem, regulating nociceptive processing at the spinal level. Thirdly, a keloid stress response (e.g., HPA-hyperfunction) provokes neurotoxic effects and maladaptations such as limited cell proliferation, volume matter reduction, and re-organisation in brain areas which are involved in pain processing (e.g., the hippocampal area) [47,48]. The adaptation to both chronic stress and to chronic pain can promote changes in the volume of grey matter [47]. Fourthly, an altered metabolic system (such as local lipid storage or the amount of cholesterol in the plasma membrane) can trigger myelination and peripheral nerve function [52] as well as pain transmission [53]. In sum, several stress-associated mechanisms are involved in the human pain response.

### 4.2. Adaptation to Relaxation Treatments in Therapy

In considering relaxation techniques in order to attenuate the aforementioned stress mechanisms, the mindfulness-based stress reduction (MBSR) program—as one stress reduction program amongst others [54]—may be a plausible treatment strategy. This structured therapy program combines elements of meditation, yoga, and physical attention. It aims to encourage the practice of body-related focused attention in daily life situations and supports the acceptance and processing of pain-associated stimuli and even negative emotions.

Its effectiveness in treating chronic pain has been reported in several different studies [55,56] and its effects are comparable to the mean effects of cognitive-behavioral therapy [57]. Regarding the pronounced reciprocal regulation of pain stimuli (see Section 2) it is relevant to note that body-related focused attention is associated with increased neural activity in the insular cortex [58,59]. Furthermore, neural activity during body-related focused attention is associated with concentration of the neurotransmitter GABA in the insular cortex, a region where body-related physiological stimuli including pain stimuli are processed [59] (see Figure 1), amongst other regions in the human brain (anterior cingulate cortex, primary/secondary somatosensory cortices, etc.). In addition, the application of mindfulness meditation was linked to a reduction in fear-avoidance behavior and improved perception of sensory information [60].

This supports the ideas of restructuring of volume and of biochemical deteriorations that affect pain patients [61]. Further, MBSR supports the regulation of emotions (e.g., in the medial prefrontal cortex) and can reduce stress and anxiety, which in turn can interfere with the pain response in humans. Persons who are exposed to traumata or high frequency stressors can show a limited stress response on the HPA axis. In this case pain stimuli are not associated with increased intrinsic brain activity, but rather deficient pain inhibition, which in turn is linked to increased pain sensitivity [48].

## 5. Interaction between Mental Stress and Physical Stress (MS and PS)

Having briefly reviewed the individual effects of physical stress (e.g., exercise), mental stress (e.g., psychosocial stress) and the potential neurobiological consequences in the genesis and treatment of chronic pain, it is relevant to look at the interaction of two treatment forms (motor control exercise, mindfulness-based stress reduction) within multimodal programs in more detail. Although the Cochrane reviews report a higher effectiveness of multimodal treatments in comparison to unimodal physical or cognitive-behavioural treatments [34,62,63,64], it is still unclear if this combination must be oriented to the suggested effects of each treatment form itself.

Numerous studies point out that moderate exercise modulates the activity of the HPA axis, leading to a reduced degree of stress responsiveness, improved ANS regulation, and altered HPA axis function resulting in a reduced allostatic load. More evidence accumulates when considering the positive effects of moderate exercise on brain development (e.g., cell proliferation), as well as the moderation of depressive symptoms, anxiety, and cognitive problems. On the contrary, high intensity exercise does not exert these positive influences; rather, high intensity exercise induces a delayed feedback mechanism in the HPA axis (stopping glucocorticoid release) and the allostatic load increases. Under these conditions, the likelihood of the successful development of an EIH or an acute induced stress analgesia decreases. It is obvious that an interaction between physical and mental stress also affect therapy programs for pain patients.

For example, individuals with a hyperfunction of the HPA axis show increased pain sensitivity during and after a “normal” exercise dose, and reduced pain symptoms only during or after a mild exercise dose. In addition, they show disturbed affect regulation in response to applied pain stimuli [65]. The choice of the most suitable exercise dose must therefore be aligned to the individual’s MS status—in this case the HPA function—which can be difficult to achieve. In the current example a multimodal program, starting with MBSR training followed by motor control training, would probably generate the most beneficial effects. The choice of an optimal combination of different treatment forms may be important, but has been completely neglected in recent programs and could serve to explain the reported short-term effects of exercise treatments [33]. However, there is a lack of scientific evidence for such claims, as selection tools for the classification of appropriate exercise intensity or behavioural treatments are still missing. Recently, a novel diagnostic tool was developed which allows a stratified treatment allocation to additional biopsychosocial treatment in combination with exercise treatment [66]. This screening tool identifies MS as well as affective-cognitive aspects of pain (including anxiety and emotion) while respecting the individual physical activity status. As the evidence suggests that these factors may be important to explore further, this tool for individual stratified treatment allocation could be helpful in planning future and optimal treatments.

## 6. Conclusions

This paper discusses possible pathways between physical and mental stress and potential neurobiological consequences in the genesis and treatment of chronic pain. Therapeutically relevant targets, such as pain-related neural maladaptations and pain-related mechanisms between exercise and relaxation techniques, are presented and discussed. By highlighting some evolving concepts, promising research directions in the prevention and treatment of chronic pain may be established.

## Abbreviations

ANS	autonomic nervous system
CNS	central nervous system
DLPFC	dorsolateral prefrontal cortex
EIH	Exercise-induces hypoalgesia
fMRI	functional magnetic resonance imaging
GABA	γ-aminobutyric acid
HPA	hypothalamic–pituitary–adrenal axis
HPG	hypothalamic–pituitary–gonadal axis
HPP	hypothalamic–pituitary–prolactin axis
HPS	hypothalamic–pituitary–somatotropic axis
HPT	hypo-thalamic–pituitary–thyroid axis
LBP	low back pain
CLBP	chronic low back pain
MBSR	mindfulness-based stress reduction program
MPFC	medial prefrontal cortex
MS	mental stress
PS	physical stress
rsfMRI	resting-state functional magnetic resonance imaging
SMN	sensory-motor neural network
sMRI	structural magnetic resonance imaging
SMT	sensory-motor training
SNRIs	selective noradrenaline reuptake inhibitors
SSRIs	selective serotonin reuptake inhibitors
NSAIDs	nonsteroidal anti-inflammatory drugs
